# How Do Human Cells React to the Absence of Mitochondrial DNA?

**DOI:** 10.1371/journal.pone.0005713

**Published:** 2009-05-28

**Authors:** Rossana Mineri, Norman Pavelka, Erika Fernandez-Vizarra, Paola Ricciardi-Castagnoli, Massimo Zeviani, Valeria Tiranti

**Affiliations:** 1 Unit of Molecular Neurogenetics – Pierfranco and Luisa Mariani Center for the study of Mitochondrial Disorders in Children, IRCCS Foundation Neurological Institute “C. Besta”, Milan, Italy; 2 Department of Biotechnology and Bioscience, University of Milano-Bicocca, Milano, Italy; 3 Singapore Immunology Network (SIgN), Agency for Science, Technology and Research (A*STAR), Singapore, Singapore; Victor Chang Cardiac Research Institute (VCCRI), Australia

## Abstract

**Background:**

Mitochondrial biogenesis is under the control of two different genetic systems: the nuclear genome (nDNA) and the mitochondrial genome (mtDNA). The mtDNA is a circular genome of 16.6 kb encoding 13 of the approximately 90 subunits that form the respiratory chain, the remaining ones being encoded by the nDNA. Eukaryotic cells are able to monitor and respond to changes in mitochondrial function through alterations in nuclear gene expression, a phenomenon first defined in yeast and known as retrograde regulation. To investigate how the cellular transcriptome is modified in response to the absence of mtDNA, we used Affymetrix HG-U133A GeneChip arrays to study the gene expression profile of two human cell lines, 143BTK^−^ and A549, which had been entirely depleted of mtDNA (ρ° cells), and compared it with that of corresponding undepleted parental cells (ρ^+^ cells).

**Results:**

Our data indicate that absence of mtDNA is associated with: i) a down-regulation of cell cycle control genes and a reduction of cell replication rate, ii) a down-regulation of nuclear-encoded subunits of complex III of the respiratory chain and iii) a down-regulation of a gene described as the human homolog of *ELAC2* of *E. coli*, which encodes a protein that we show to also target to the mitochondrial compartment.

**Conclusions:**

Our results indicate a strong correlation between mitochondrial biogenesis and cell cycle control and suggest that some proteins could have a double role: for instance in controlling both cell cycle progression and mitochondrial functions. In addition, the finding that *ELAC2* and maybe other transcripts that are located into mitochondria, are down-regulated in ρ° cells, make them good candidates for human disorders associated with defective replication and expression of mtDNA.

## Introduction

Mitochondria are double-membrane cytoplasmic organelles that house a number of fundamental cellular pathways and functions, including, the aerobic synthesis of ATP [1]; the biosynthesis of heme, ketones, and uridine [2], calcium trafficking, and control of apoptosis [3]. Since reactive oxygen species (ROS) are by-products of respiration, mitochondria are also the major source of ROS, being equipped with protective systems to prevent damage determined by these harmful compounds.

Mitochondria have their own DNA [4]: the human mitochondrial genome (mtDNA) is a 16.6 kb double-stranded circular DNA molecule encoding 13 of the approximately 90 subunits that form the respiratory chain, the remaining ones being encoded by the nuclear genome (nDNA). These latter proteins are synthesized in the cytoplasm and imported into mitochondria where they are assembled, together with the mitochondrially encoded subunits, to form the respiratory chain complexes of the inner mitochondrial membrane [5].

While important factors involved in mtDNA expression and replication have been identified in humans, little is known about the molecular mechanisms regulating the nuclear-mitochondrial cross-talk. Eukaryotic cells are able to monitor and respond to changes in mitochondrial function through alterations in nuclear gene expression, a phenomenon first defined in yeast and known as retrograde regulation [6]. To investigate the nuclear response to impairment of mitochondrial function, a commonly applied method involves the use of so-called ρ° cell lines, i.e. cells that have been completely depleted of mtDNA by prolonged exposure to sub-lethal doses of ethidium bromide (EthBr). For instance, Delsite et al. [7] used spotted radioactive microarrays to compare the transcriptome of a human breast cancer cell line and its ρ° derivative and found deregulation of a number of different genes involved in pathways as diverse as cell signalling, metabolism, cell growth and differentiation. Behan et al. [8] used Affymetrix technology to study the nuclear gene expression response to the lack of mitochondrial DNA in ρ° and ρ^+^ (i.e. non-depleted parental) Nawalma cells. According to their analysis the most affected cellular functions were exemplified by genes involved in hypoxic response, glycolysis and fatty acid oxidation and genes encoding mitochondrial ribosomal proteins, transport channels and tRNA synthases. More recently, Magda et al. [9] used Affymetrix microarrays to compare ρ° and ρ^+^ A549 lung cancer cells grown either in culture or as xenografts in nude mice. Results from that study showed that mtDNA levels regulated the expression of genes involved in glucuronydation, tRNA synthetase, immune surveillance and peroxisomal lipid metabolism. Although these studies proved valuable insights into the complexity of the nuclear response to the absence of mtDNA, their different results also emphasize the difficulty in identifying common signatures of mtDNA depletion across various model systems. The partially non-overlapping results reported in above-cited studies may be attributed either to the use of different microarray platforms and analysis methods or to the use of different cell lines and growth conditions. Cell-type specific effects had already been reported by quantitative transcription analysis of a selected number of nuclear genes across two different human ρ° cell lines, their ρ^+^ counterparts, and fibroblasts from a patient affected by Kearns-Sayre syndrome (KSS) [10]. Furthermore, growth conditions proved to have a higher influence on gene expression profiles than the effect of mtDNA depletion itself [9].

To identify which nuclear transcripts are subjected to retrograde regulation in human cells regardless of the specific cell type, we performed a comparative microarray analysis of global RNA expression profiles in two immortal human cell lines and in their ρ° cell derivatives. Using a stringent statistical approach, we identified 191 genes the expression of which was significantly and consistently different in both ρ° cell lines compared with their parental cell lines.

## Results and Discussion

### Gene expression profiling of human ρ° cell lines

**Figure 1:**
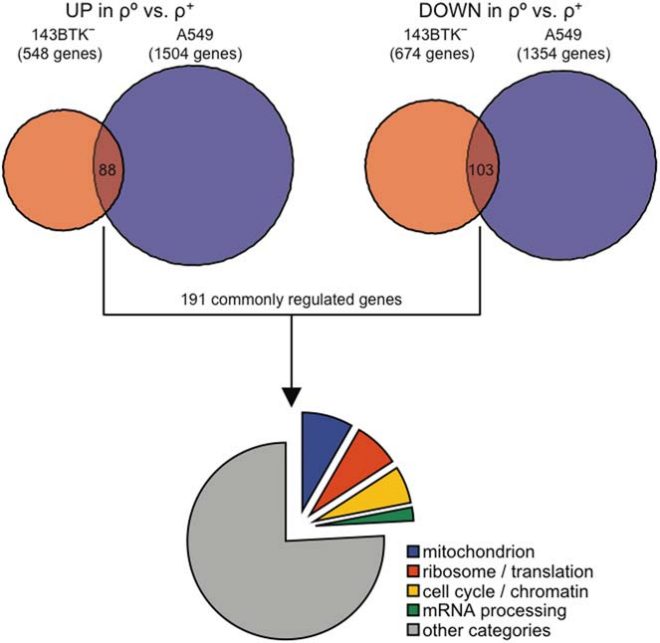
Microarray data analysis strategy. A schematic overview of the strategy used to identify genes commonly up- or down-regulated in ρ° cells is given (see Materials and Methods for details).

In order to minimize cell line-specific effects on nuclear transcription due to EthBr treatment, we compared the expression profiles obtained from two different cell lines, 143BTK^−^ osteosarcoma cells and A549 lung adenocarcinoma cells, to that of their mtDNA-less (ρ°) derivatives. The latter were generated with no clonal selection (see Materials and Methods), in order to dilute any possible effect due to chromosomal aneuploidy, which can occur in cancer-derived cultured cells. Three independent biological replicates were used for each cell type. The method adopted to identify differentially expressed genes takes into account the variance-versus-mean dependence that exists in microarray data, which typically follows a power law [11]. Using the Power Law Global Error Model (PLGEM) analysis method, selection of significant changes in gene expression is intrinsically more stringent for transcripts with very low expression levels, than for transcripts with high expression levels [11]. Due to high noise of microarray data and to the relatively small sample size in comparison to the number of analyzed genes, our approach was mainly aimed at limiting the amount of false predictions. A total of 191 genes (Table S1) were identified as differentially expressed in both cell lines: 88 genes in common were up-regulated while 103 in common were down-regulated (Figure 1). Only ∼30 genes were expected to be selected by chance using this statistical technique (see Materials and Methods for details).

In order to determine whether our gene cohort was significantly enriched in gene products associated with specific molecular functions, sub-cellular compartments or biological processes, we analyzed the functional annotations of the 191 differentially expressed transcripts by using their associated Gene Ontology (GO) terms. The most significantly enriched GO terms were used as a guide to manually assemble a functional classification of the differentially expressed genes, grouping together terms with similar biological function. Four main functional categories were identified in this way (Figure 1 and Table S2): genes encoding mitochondrial proteins (16 entries), genes involved in protein synthesis (14 entries), genes involved in cell cycle and chromatin structure (12 entries) and genes involved in mRNA processing (4 entries).

### Validation of microarray data by real-time PCR and Western blot

**Figure 2:**
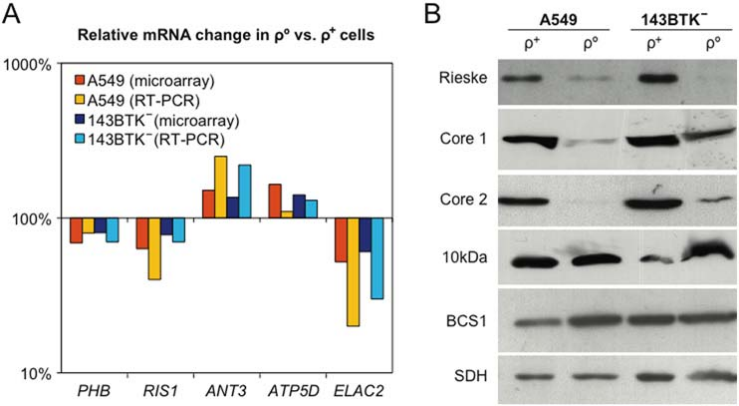
Validation of microarray data. (A) Comparison between relative mRNA changes predicted by microarray and those verified by RT-PCR analysis, in either A549 or 143BTK− cells. Bars represent average relative expression changes of the genes indicated at the bottom, comparing ρ° cells vs. ρ+ controls across three biological replicates. (B) Western blot analysis on ρ+ and ρ° cell lysates of complex III subunits (Rieske, Core 1, Core2, and 10 kDa subunits) and of the assembly factor BCS1L. To normalize for the protein content in each lane an SDH specific antibody was used as a mitochondrial standard.

We confirmed the results obtained by microarray analysis by quantitative RT-PCR (real-time PCR) on a limited number of genes. The absence of mtDNA and mitochondrial specific transcripts was demonstrated in ρ° cells by quantitative PCR (data not shown). As reported in Figure 2A, five gene-products whose differential expression passed the statistical test (RIS1, ANT3, PHB, ATP5D, ELAC2) in microarray experiments, also showed significantly different expression levels by RT-PCR. On the contrary, expression of genes involved in mtDNA transcription (such as TFAM) and replication (such as SSBP1, POLG1 and PEO1) that did not pass the statistical test, for instance because of a “No Change” call in either one of the microarray replicates, was also unchanged by real-time PCR (data not shown).

The Rieske iron-sulfur protein, a component of the complex III of the respiratory chain, was found significantly down-regulated by microarray analysis in both ρ° cell lines. Marked reduction of the corresponding protein was confirmed by Western blot analysis on SDS PAGE (Figure 2B). Conversely, transcripts of complex III 10 kDa subunit and complex III assembly factor BCS1L, were unchanged in the microarray data and the corresponding proteins were found unchanged by Western blot analysis (Figure 2B).

### Differential effects on complex V and complex III in cells lacking mtDNA

Table S2 reports 16 differentially expressed nuclear genes coding for mitochondrial proteins. Both “up” and “down” changes were detected in ρ° vs. ρ^+^ cells. For instance, the transcripts of ATP5D, which is part of the F1 particle of the ATP synthase (complex V), and isoform 3 of the adenine nucleotide translocator (ANT3) were both up-regulated (+30–40%) by microarray and RT-PCR analysis (Figure 2A). ρ° cells retain both an aurovertin-sensitive ATPase activity, provided by the F1 particle, and an adenine nucleotide translocation activity, carried out by the three ANT isoforms [12]. These activities are essential to maintain the mitochondrial membrane potential, prevent osmotic swelling of the organelle and avoid apoptosis due to cytochrome c release [13].

As already mentioned, an opposite effect was observed in genes encoding subunits of the complex III, for instance, transcription of the Rieske iron-sulphur protein [14] was down by 60–70%. This phenomenon was specific because the expression of other genes encoding for respiratory chain complexes was unaltered. Complex III (Ubiquinol cytochrome c reductase), is a 250 kDa homodimer, each monomer being composed of 12 subunits, including the mtDNA encoded cytochrome b. The Rieske protein plays a major role in complex III function; by transferring electrons from ubiquinol to cytochrome b and from cytochrome c1 to cytochrome c. Western blot confirmed a profound reduction of the Rieske protein in both ρ° cell types (Figure 2B). Although the mRNA levels of three other components of complex III (i.e. Core 1, Core 2 and the 10 kDa subunit) were not significantly changed by microarray analysis, Core 1 and Core 2 were significantly decreased at the protein level whereas the 10 kDa subunit was not (Figure 2B). However, it has to be considered that the level of mRNA expression does not directly correlate with the amount of the corresponding protein. This is particularly true for the OXPHOS apparatus, since the stability of each single complex is strongly influenced by the stability of the others. Although we did not carry out Western blot analysis on all the nuclear-encoded protein subunits forming the OXPHOS complexes, we observed that the absence of assembled complex III in ρ° cells is associated with degradation of some of its protein components. On the other hand, the BCS1L protein that is responsible for the insertion of the Rieske protein into the pre-complex III, as one of the last steps in complex III assembly [15], is present in comparable amounts in both ρ° and ρ^+^ cells according to microarray data and Western blot analysis (Figure 2B). This indicates that the lack of structural complex III subunits does not determine a decreased production of the BCS1L protein, suggesting either a mechanism of compensation in the absence of complex III subunits or an additional function for BCS1L unrelated to complex III assembly.

Complex III is the major site of ROS production during hypoxia and plays a central role in the oxygen-sensing pathway [16], [17]. ROS have been demonstrated to modulate respiration in mammals by regulating several checkpoints energy pathways and mtDNA homeostasis. Since respiration-incompetent ρ° cells fail to generate ROS, an attractive hypothesis prompted by our observation is that ROS control on respiration could be mediated, at least in part, by modulating the expression of the Rieske protein and the activity of complex III.

### Absence of mtDNA delays cell cycle progression

**Figure 3:**
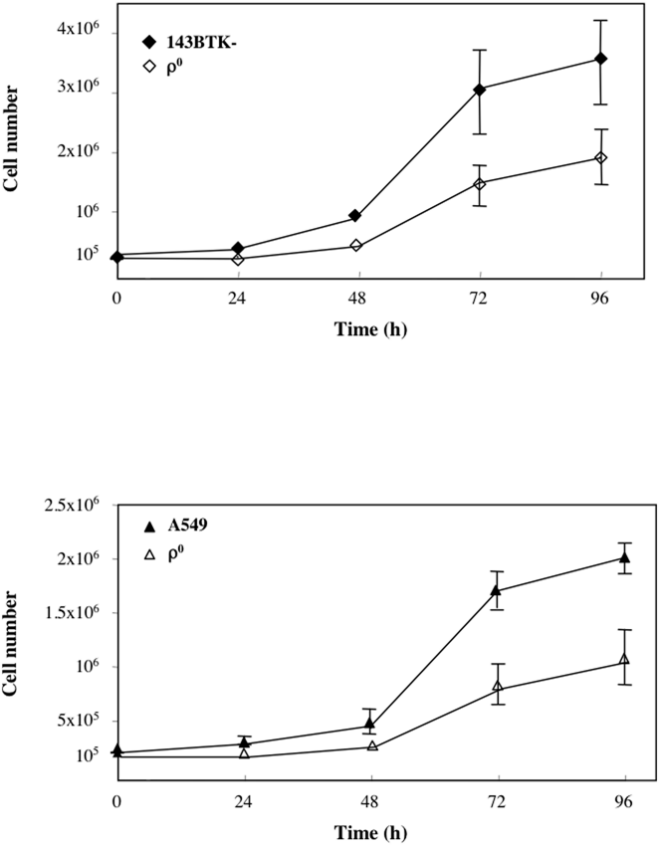
Reduced cell proliferation of ρ° cells Cell proliferation curves of 143BTK− (A) and A549 cells (B) are shown. Closed symbols, ρ+ cells; open symbol, ρ° cells. Points and error bars respectively represent the mean and the standard deviation of three independent experiments.

We observed a generalized down-regulation of genes involved in the control of cell cycle (Tables S1 and S2), including H2AFZ and H2AFX, two members of the histone family H2A, cyclin E2, and Histone deacetylase 1 (HDAC1), which play an important role in both transcriptional regulation and cell cycle progression [18]. Interestingly, down-regulation of HDAC1 can explain that of cyclin E2, since it is known that HDAC1 inhibition alters the expression of several genes including cyclin E [19]. Recent studies report for mitochondria a role in the regulation of cell growth and differentiation [20], [21], [22]. To investigate whether down-regulation of cell-cycle genes could affect growth and proliferation rate, cells were counted daily for 5 days. The doubling time of ρ° cells derived from both 149BTK^−^ and A549 cell lines was significantly longer (30 and 39 hrs) than that of their parental ρ^+^ cells (23 and 29 hours (Figure 3). Our results are in agreement with recent data by Magda et al. on A549 cells [9] and by Schauen et al. obtained in ρ° HeLa cells [22].

A link between cell-cycle control and mitochondrial biogenesis is suggested by the observation that transcripts for two mitochondrial proteins, prohibitin (PHB) [23] and C1QBP/p32 [24] were both down regulated in ρ° cell lines. PHB acts as a chaperone protein, is essential for normal mitochondrial biogenesis in *C. elegans*, [25], and plays a role as a regulator of cell-cycle progression [26], [27], [28]. Recently Merkwirth and Langer demonstrated that mitochondrial prohibitin complexes control cell proliferation, cristae morphogenesis and the functional integrity of mitochondria [29]. C1QBP/p32 is mainly localized to the mitochondrial matrix [30], but a small fraction is also present in the nucleus, which suggests a role for p32 as a factor mediating nucleus-mitochondrial crosstalk. Nuclear p32 is believed to be involved in the control of cell growth and cell cycle via the MAP kinase pathway [31].

Taken together, these data suggest the existence of a cross-talk between the nuclear and the mitochondrial genome that slows down cell cycle progression in case of mitochondrial defects. Schauen et al. ruled out energetic or metabolic impairment as the direct cause of the reduced cell proliferation of ρ° HeLa cells, whereas they attributed it to a reduction of ROS generation and p21^CIP1/WAF1^ expression. Future work is warranted to elucidate whether this, or an alternative mechanism, is implicated in the down-regulation of cell cycle control factors observed by us.

### Identification of a novel mitochondrial transcript down-regulated by lack of mtDNA

Among the poorly characterized genes found to be down-regulated in ρ° cells, shown by microarray analysis, and confirmed by RT-PCR, we focused our attention on *ELAC2* [32], which was first identified as a prostate cancer susceptibility gene [33]. Elac2 is the human homolog of tRNase Z in *E. coli*, which is responsible for the endonucleolytic cleavage of the 3′ end of tRNAs.

**Figure 4:**
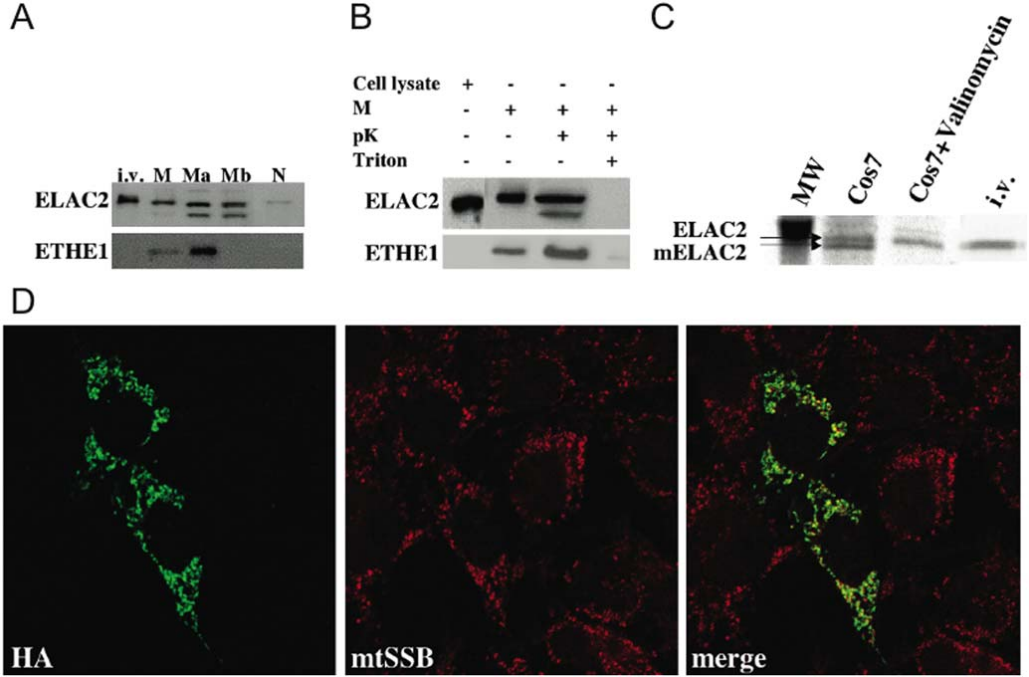
Mitochondrial localization of Elac2. (A) Sub-organellar localization of Elac2 by western-blot analysis on mitochondria isolated from HeLa cells. Specificity of antibody reactivity was proved by the CRM obtained using an in vitro translated (i.v.) Elac2. A CRM was present in mitochondria (M), in the mitochondrial matrix (Ma) and membranes (Mb), and barely detectable in the nucleus (N). ETHE1 antibody was used as a mitochondrial matrix control protein. (B) Proteinase K protection assay by western-blot analysis on freshly isolated mitochondria from HeLa cells. Lane 1, cell lysate; lane 2, intact mitochondria; lane 3, mitochondria treated with 100 µg/ml of proteinase K; lane 4, mitochondria treated with 0.5% of Triton-X100. (C) Mitochondrial in vivo import of HA-tagged human Elac2 N-terminal polypeptide expressed in Cos-7 cells. Lane 1: marker 46 kDa (MW). lane 2: immunoprecipitation of total radiolabeled proteins. lane 3: immunoprecipitation of total radiolabeled proteins after the addition of valinomycin that determines the elimination of the mitochondrial ΔΨ: as a consequence, mitochondrial import is abolished. lane 4: immunoprecipitation of in vitro translation product (i.v.). Arrows indicate the Elac2 precursor (Elac2) and the mature form (mElac2). (D) Confocal immunofluorescence on Cos7 cells. The green fluorescence corresponds to Elac2HA-specific immunoreactivity. The red fluorescence corresponds to immunoreactivity specific to mtSSB. The two immunofluorescence patterns overlap, as shown by confocal merge. Magnification: 40×.

The Elac2 N-terminus scores high for mitochondrial targeting when analyzed by *ad hoc* prediction softwares (Mitoprot, Predotar, TargetP, PSORT), consistent with the idea that Elac2 might be imported into mitochondria. To test this hypothesis experimentally we carried out Western blot analysis on sub-cellular fractions (Figure 4A–B), by using a commercially available anti-Elac2 polyclonal antibody. The specificity of the Elac2 antibody was tested by immuno-detection of an *in-vitro* translated product corresponding to the human *ELAC2* cDNA (Figure 4A, lane i.v.). An Elac2 specific CRM (Cross-Reacting Material), was present in isolated mitochondria and in mitochondrial fractions (matrix and membrane in Figure 4A), but was also detected, although at low level, in the nucleus. We also demonstrated that Elac2 is located inside the organelle since treatment with proteinase K (pK) failed to digest the protein in intact mitochondria, while it underwent complete digestion after solubilization of mitochondrial membranes with Triton X-100 (Figure 4B).

In order to understand if the N-terminus of Elac2 contained a cleavable mitochondrial targeting peptide, we generated a recombinant short version of Elac2 flagged with the HA epitope on the C-terminus (see Methods). This construct was used to transfect Cos7 cells (Cos-7^Elac2-HA^) and to perform in vivo immunoprecipitation (Figure 4C) and immunofluorescence studies (Figure 4D).

Using an anti-HA antibody, two ^35^S radiolabeled protein bands were immunoprecipitated from a lysate of Cos-7^Elac2-HA^ cells. The size of the higher molecular weight band (Figure 4C) corresponds to a protein of 36.5 kDa, which we believe is the mitochondrial precursor protein, while the lower molecular weight band of approximately 34.7 kDa corresponds to the mature mitochondrial Elac2 (mElac2) species, generated by enzymatic cleavage of the aminoterminal leader peptide from the precursor (Figure 4C). Since import is dependent on the presence of the mitochondrial transmembrane electrochemical gradient, only the band corresponding to the precursor was present after incubation with valinomycin, a mitochondrial ionophore that destroys the mitochondrial membrane potential. The size difference between the two bands suggests that the leader peptide encompasses the first 16 aminoacid residues, as predicted by TargetP. The mitochondrial localization of Elac2 was further confirmed by the coincidence of the Elac2-HA immunofluorescence pattern with that of mtSSB, a mitochondrial matrix protein (Figure 4D).

Our results, and the similarity of Elac2 with tRNAse Z, suggest for this protein a function in mitochondrial tRNA processing. This hypothesis has been confirmed for TRZ1, the yeast homologue of mammalian Elac2 [34] and for the RNaseZ of Drososphila melanogaster [35]. The Elac2 ortholog is up-regulated during mitosis in *S. cerevisiae* [36]. Therefore, down-regulation of Elac2 expression may be involved in the delay of cell cycle and reduced proliferation rate observed in ρ° cells. In addition, Elac2 over-expression may alter mitotic progression in HeLa cells possibly by interacting with the γ-tubulin complex [37]. Finally, Elac2 has been located also in the nucleus [38], suggesting for this protein additional functions, including a role in nuclear-mitochondrial cross-communication.

### Additional considerations

Depletion of mtDNA has been correlated with chromosomal instability in 143B cell lines [39]. Although we did not perform a detailed karyotyping of the cell lines used in the present study, there is a straightforward way to check for possible chromosome copy number changes from gene expression microarray data. On average, expression of genes on aneuploid chromosomes has been shown to scale linearly with chromosome copy number in both yeast and mammalian cells [40], [41]. However, the average gene expression change was similar across all chromosomes in both our 143BTK^−^ ρ° cells and our A549 ρ° cells compared to their parental counterparts (Figure S1). Looking at gene expression ratios as a function of chromosomal coordinate, we also did not detect any significant segmental copy number changes (data not shown). Although this analysis cannot rule out the possibility for chromosomal translocations, it excludes that mtDNA depletion induced stable and clonal aneuploidies in our cell lines. It is still formally possible, though less likely, that the cell populations used for this microarray analysis were composed of a heterogeneous mixture of cells with different aneuploidies that could have cancelled each other out (i.e. for every cell that gained a particular chromosome there could have been another cell that lost that same chromosome). Either way, aneuploidies would not have significantly affected the gene expression profiles described in this report.

Due to the remarkable contribution of cell-type specific effects on the transcriptional response of cells to mtDNA depletion, it was difficult for us to compare our gene expression signatures with previously reported microarray data obtained from human ρ° cell lines. The closest match we were able to find is with the report from Magda et al. [9], which used a cell line similar to one of ours (i.e. A549) and the same microarray platform (i.e. Affymetrix HG-U133A). Of the 131 genes reported to be up-regulated in A549 ρ° vs. ρ^+^ cells in their study, 24 (∼18%) were found also among the up-regulated genes in the same cells in this study. Of the 145 down-regulated genes in their report, 51 (∼35%) were found also in ours. The relatively low overlap could be explained by the different cell culture conditions used to grow the cells (different growth medium and glucose concentration), the different time span given to the cells to adapt to the ρ° status (freshly derived ρ° cells in their case, well established ρ° lines in ours) or to the different statistical analysis methods used to detect the differentially expressed genes.

To our surprise, no genes involved in replication and transcription of mtDNA, were present in the output of our analysis. This could be due to the fact that: 1) these genes could have an expression level that was below the threshold of sensitivity of the method, or 2) these genes behave differently either in the different cell lines or in one of the three replicates. However, since we analyzed two different cell lines that were subjected to the same treatment to eliminate the mtDNA and that were maintained in culture using exactly the same growth medium and conditions, the genes selected by this analysis could be considered as a *bona fide* set of genes differentially expressed in reply to the absence of mtDNA.

### Conclusions

Absence of mtDNA in human cells triggers a complex nuclear gene expression response, particularly as regarding genes involved in cell cycle control and mitochondrial functions. Our analysis also suggests that some genes could have a double role in both mitochondrial functions and in other cellular processes. For instance, prohibitin (PHB), C1QBP/p32 and Elac2 are part of the mitochondrial proteome and are also involved in the control of cell cycle progression. Likewise, BCS1L – which is clearly involved in complex III assembly – has also been suggested to play a role in iron metabolism and possibly other metabolic pathways [42].

In prohibitin knock-down cells, the mtDNA copy number is decreased [43], suggesting that this protein is a candidate for human conditions associated with mtDNA depletion [44]. Likewise, Elac2 could be involved in mitochondrial disorders associated with defects of mtDNA transcription and translation.

## Materials and Methods

### Cell lines and EthBr treatment

All cells were maintained in a 5% CO_2_ humidified atmosphere at 37°C. 143BTK^−^ osteosarcoma cell line and A549 lung carcinoma cell line (a kind gift of Dr. Ian Holt), were grown for 3–4 weeks in Dulbecco's modified Eagle medium (DMEM), high-glucose (Euroclone), supplemented with 10% Fetal Calf Serum (FCS), 1 mM NaPyruvate and 50 µg/ml uridine. To generate ρ° cells we added 50 ng/ml ethidium bromide (EthBr) to the growth medium, for a period of 3 weeks. No clone selection was performed but the cells were trypsinised and pooled together. One week after the removal of EthBr, absence of mtDNA was checked in ρ° cell lines by either PCR or by Southern blot. 143BTK^−^, A549 and their ρ° derivatives were stored in liquid nitrogen until needed for experiments. They were then revived and grown for around 10 passages to isolate RNA for microarray analysis. Absence of mtDNA was checked by PCR before each experiment.

### Cell growth

Cells were plated at a density of 2,000 cells per cm^2^ and grown in complete DMEM medium. At selected intervals cells were harvested by trypsinization and counted using the Trypan Blue vital staining. Growth rate was calculated by determining the duplication time [45].

### cRNA preparation and microarray hybridization

RNA samples for microarray analysis were prepared according to the manufacturer's recommendations. Briefly, total RNA was isolated from 8×10^6^ cells using the Qiagen RNeasy Mini kit (Qiagen); 12 µg of total RNA was then used to generate double-stranded cDNA using an oligo (dT24) primer containing a T7 polymerase recognition sequence. The cDNA was then used as a template for in vitro transcription reaction (BioArray High Yield RNA transcript labelling kit ENZO) using biotinylated CTP and UTP to produce labelled cRNA. The cRNA was then fragmented and hybridized to HG-U133A GeneChip arrays (Affymetrix, Santa Clara, CA), which contain 22,283 probe sets designed to interrogate the expression of more than 13,000 unique human genes. Hybridized chips were then washed and stained with streptavidin-phycoerytherin and scanned with an Affymetrix GeneChip scanner. Resulting image files were processed by Affymetrix Microarray Suite 5.0 (MAS5) to compute probe set-specific expression values, e.g. Signal, Detection, etc. Three independent RNA extractions were performed for each cell lines (both for parental and for ñ° derivatives), reaching a total of 12 microarrays. The microarray dataset was deposited in the public Gene Expression Omnibus (GEO) database (www.ncbi.nlm.nih.gov/geo/) under accession number GSE14900.

### Data pre-processing and identification of differentially expressed genes

The data set containing expression values for 22,283 probe sets and 12 samples was analyzed inside the R environment [46] mainly using packages from the BioConductor project [47]. First, a “quantile normalization” [48] was applied to each of the two subsets of data corresponding to a given cell line, independently. Then, genes called “Absent” by MAS5 across all six samples of a given cell line were removed, leaving 14,012 and 13,096 probe sets in the 143BTK^−^ and the A549 dataset, respectively. The intersection between these two sets was of 12,031 probe sets. Microarray data from the three biological replicates available for each ρ° cell line were then compared to the three biological replicates of the corresponding parental cells using the BioConductor package “plgem” [11]. A PLGEM was fitted on an independent data set, containing Affymetrix HG-U133A data from eight distinct individuals, which had been pre-processed in the same way as the data reported in the present work. Resulting model parameters were used to calculate model-based signal-to-noise statistics when comparing ρ° cells versus parental cells. Genes were selected as differentially expressed in a particular cell line, if their observed signal-to-noise ratio was more extreme than the 5% most extreme statistics values observed during a series of 1,000 random re-samplings performed on the set of data set used to fit the model.

Though the two differential expression analyses performed independently on 143BTK^−^ and on A549 cells were run using quite relaxed stringency parameters (per-gene significance level = 0.05), a reduction of the false positive rate is expected when combining results from two independent analyses. Despite the impossibility to report an accurate p-value of the combined result, it is possible to speculate the following. In the case that not a single gene was actually differentially expressed, ∼700 genes ( = 14,012×0.05) and ∼655 genes ( = 13,096×0.05) would be expected to be selected by chance alone in the 143BTK^−^ and the A549 dataset, respectively. Instead, 548 were selected as up-regulated and 674 genes were selected as down-regulated in the 143BTK^−^ dataset, whereas 1,504 were up- and 1,354 were down-regulated in the A549 dataset (Figure 1A). Under the same null hypothesis, and considering a complete independence between the two analyses, the total number of false positive genes in the combined result should drop down to ∼30 ( = 12,031×0.05×0.05), while in fact 88 genes were found as commonly up-regulated and 103 as commonly down-regulated in the two cell lines (Figure 1A).

### Functional categories enrichment evaluation

The list of 191 probe sets was first converted into the corresponding 180 unique Entrez Gene IDs [49] and then analyzed with the function “GOHyperG” from the BioConductor package “GOstats” that uses the hypergeometric distribution to evaluate enrichment of particular Gene Ontology functional categories [50]. An independent analysis was performed for each of the three ontologies currently supported by the GO project, i.e molecular function, biological process, and cellular component [51].

### Quantitative RT-PCR

Total RNA was prepared from 8×10^6^ cells using the RNeasy kit (Qiagen). Superscript reverse transcriptase II (Invitrogen), was used to produce cDNA starting from 12 μg of total RNA. RT-PCR was carried out using an Applied Biosystem Inc. (ABI) Prism 7000 Sequence Detection real-time system in a two-step reaction. Primers for the genes of interest are reported in Table 1 and were designed using the ABI Prism Primer Express (2.0) software. All primers were checked by BLAST search against the mRNA database to ensure, specific homology, only to the gene of interest. Each PCR reaction was performed in triplicate in 50 µl with 600 µM (final concentration) each of the forward and reverse primers, and 25 µl of 2× SYBR green PCR Master Mix (ABI) with the following profile: one cycle at 50°C for 2 min, one cycle at 95°C for 10 min, and then 40 cycles of 95°C for 15 s, 60°C for 1 min (two-step protocol). We chose as the endogenous control the glyceraldehyde-3-phosphate dehydrogenase (GAPDH) gene.

### Western blot analysis and antibodies

Approximately 2×10^6^ cells were trypsinized, pelleted, sonicated, and solubilized as previously described. SDS-polyacrylamide gel of 100–200 µg protein/lane, and Western blot analysis were performed as described [52]. An affinity purified polyclonal antibody against BCS1L was obtained from ProteinTech Group, Inc (Chicago). The monoclonal antibodies against RIS1, Core1, and Core2 were from Invitrogen. The monoclonal antibody against the 10 kDa subunit was from MitoSciences. In order to check for equal amount of proteins for all samples, the blot was stripped and re-probed with an SDH specific polyclonal antibody (Invitrogen). A mouse monoclonal antibody against the haemoagglutinin epitope of the influenza virus (anti-HA) was from Roche. An affinity purified polyclonal antibody against ELAC2 was from ProteinTech, while the polyclonal antibody against Ethe1 was raised in rabbit by Neosystem [52].

### Mitochondrial targeting

Immunofluorescence (IF) was carried out on coverslip-plated cells. Cells were fixed and incubated with a polyclonal antibody specific to mtSSB and with a monoclonal anti-HA antibody (Roche). Fluorescent-dye conjugated secondary antibodies were used as previously described [53]. IF was visualised using a confocal microscope (Biorad). *In vivo* import was performed as described [52].

### Cell fractionation

Standard methods were used for the preparation of mitochondrial fractions in cultured cells [54]. For sub-organellar localization, freshly isolated mitochondria from HeLa cells were treated with three cycles of freezing and thawing and six sonication strokes on ice-cold water. The membrane and soluble fractions were then separated by ultracentrifugation at 100,000×g for 1 hr at 4°C. For proteinase K protection, mitochondria were incubated with 100 µg/ml of proteinase K for 20 min at 37°C, with and without the addition of 0.5% Triton X-100.

### Generation and cloning of ELAC2^HA^

A full-length human *ELAC2* cDNA clone was obtained from the RZPD consortium (clone IRALp962C132Q). The first 970 bp on the 5′ end of the human *ELAC2* cDNA were tagged on the 3′ end with the HA epitope. The following oligonucleotides were used for PCR reaction using the RZPD clone as a template. Underlined sequence corresponds to the HA epitope: 5′-CGCGGATCCGCGATGTGGGCGCTTTGCTCGCTGCTGCGGT–3′ (forward primer); 5′-CCGCTCGAGTCAAGCGTAATCTGGAACATCGTATGGGTAAATCTCACAGATGGGTTGAATG-3′ (reverse primer). Each of the 30 PCR amplification cycles was 95°C for 1 min, 70°C for 2 min. The cDNA was cloned into the plasmid vector TA-cloning TOPO II kit (Invitrogen) and sequenced. The cDNA was then inserted into the eukaryotic expression plasmid vector pcDNA3.1 (Invitrogen) using suitable restriction sites. The predicted chimeric protein has an expected molecular weight of 36.5 kDa.

## Supporting Information

Figure S1 Distribution of gene expression changes per chromosome. Gene expression log2 ratios from the 143BTK− ρ° vs. ρ+ cells (upper panel) or from the A549 ρ° vs. ρ+ cells (lower panel) were subdivided based on chromosome and their distributions shown as box-and-whisker plots. Chromosomal location was obtained from Ensembl version 53 [55] based on the NCBI 36 assembly of the human genome. Only probe sets not called always “Absent” across all samples of the corresponding GeneChip dataset and that uniquely mapped to a single locus were included in this analysis. Box-and-whisker plots were generated in R using default settings, i.e. the thick horizontal line inside the box represents the median of the distribution, and the lower and upper hinges of the box represent the first and the third quartile of the distribution, while the two whiskers extend to 1.5 times the inter-quartile range of the distribution. Outlier probe sets are plotted as circles. A red and a blue horizontal line are superimposed to the plot to show where the average gene expression ratio would be expected to be found, if the corresponding chromosome was gained once or lost once, respectively.
(5.66 MB TIF)

Table S1 191 genes showing consistent and significant differential expression in ρ° vs. ρ+ cells (in both A549 and 143BTK− cell lines). Signal, detection and ratio between ρ° and ρ+ cells are reported along with gene names and accession numbers to Entrez Gene, GenBank and UniGene. A, B and C are the three biological replicates for each condition.
(0.20 MB XLS)

Table S2 Functional classification of differentially expressed genes. Genes are grouped according to their most likely function, deduced from Gene Ontology annotations and from data available in published literature.
(0.05 MB XLS)

Table S3 Primers used in the quantitative PCR amplification analysis
(0.03 MB DOC)
