# Supplementary figures and images for: How Do Human Cells React to the Absence of Mitochondrial DNA?
Source: PLoS One. 2009 May 28;4(5):e5713. doi: 10.1371/journal.pone.0005713 (PMC2683933; doi:10.1371/journal.pone.0005713)

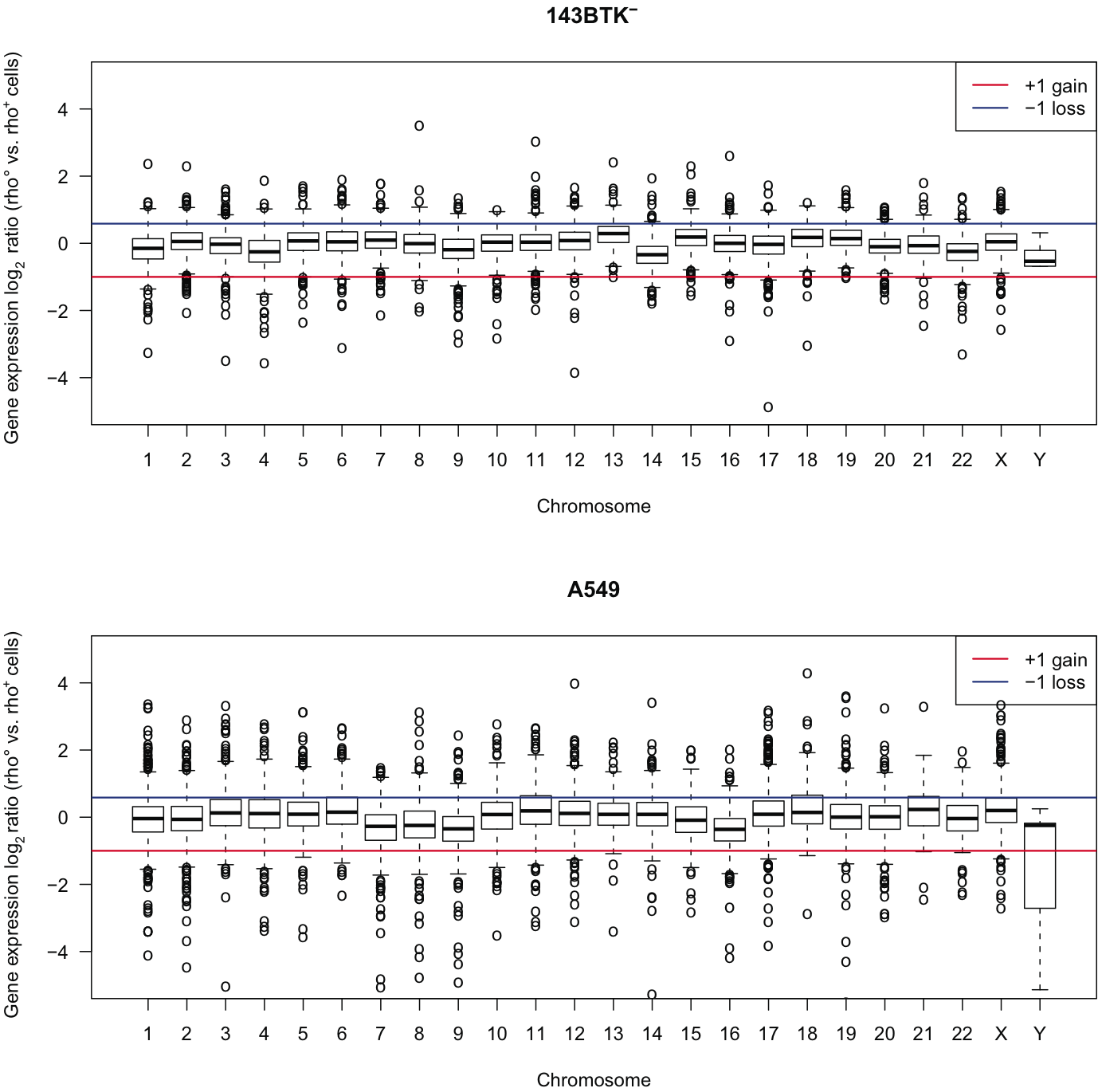

Supplement: Figure S1 — Distribution of gene expression changes per chromosome. Gene expression log2 ratios from the 143BTK− ρ° vs. ρ+ cells (upper panel) or from the A549 ρ° vs. ρ+ cells (lower panel) were subdivided based on chromosome and their distributions shown as box-and-whisker plots. Chromosomal location was obtained from Ensembl version 53 [55] based on the NCBI 36 assembly of the human genome. Only probe sets not called always “Absent” across all samples of the corresponding GeneChip dataset and that uniquely mapped to a single locus were included in this analysis. Box-and-whisker plots were generated in R using default settings, i.e. the thick horizontal line inside the box represents the median of the distribution, and the lower and upper hinges of the box represent the first and the third quartile of the distribution, while the two whiskers extend to 1.5 times the inter-quartile range of the distribution. Outlier probe sets are plotted as circles. A red and a blue horizontal line are superimposed to the plot to show where the average gene expression ratio would be expected to be found, if the corresponding chromosome was gained once or lost once, respectively. (5.66 MB TIF) [file pone.0005713.s001.tif]
