# Supplementary material for: How Do Human Cells React to the Absence of Mitochondrial DNA?
Source: PLoS One. 2009 May 28;4(5):e5713. doi: 10.1371/journal.pone.0005713 (PMC2683933; doi:10.1371/journal.pone.0005713)
Supplement: Table S3 — Primers used in the quantitative PCR amplification analysis (0.03 MB DOC) [file pone.0005713.s004.doc]

| **GAPDH** | Forward | 5'-ATGGAAATCCCATCACCATCTT-3' |
| --- | --- | --- |
|  | Reverse | 5'-CGCCCCACTTGATTTTG-3' |
| **RIS1** | Forward | 5'-GCCGTCACCCAGTTCGTTT-3' |
|  | Reverse | 5'-TTCGCCAGGGCCAACA-3' |
| **BCS1** | Forward | 5'-CTGGGCCTGGTGGCATT-3' |
|  | Reverse | 5'-CGAGCAGGGACTTCCAGTGT-3' |
| **ANT3** | Forward | 5'-TTGCCAACGTCATTCGCTACT-3' |
|  | Reverse | 5'-TGTACTTATCCTTGAAGGCGAAGTT-3' |
| **ATP5D** | Forward | 5'-TTGTGAGCAGCGGTTCCA-3' |
|  | Reverse | 5'-CCAACAACTGCACCGAAGAGT-3' |
| **PHB**  **ELAC2** | Forward  Reverse  Forward  Reverse | 5’-GCGTGGTGAACTCTGCCTTATA-3’  5’-CACGGAATCGGTCAAAGATGA-3’  5’-TTGCTCGCCTGGACAACAT-3’  5’-TAAGCCCCCAACATTAGACCA-3’ |
